# Differences in Sex and the Incidence and In-Hospital Mortality among People Admitted for Infective Endocarditis in Spain, 2016–2020

**DOI:** 10.3390/jcm11226847

**Published:** 2022-11-20

**Authors:** Jose M. De Miguel-Yanes, Rodrigo Jimenez-Garcia, Javier De Miguel-Diez, Valentin Hernández-Barrera, David Carabantes-Alarcon, Jose J. Zamorano-Leon, Concepción Noriega, Ana Lopez-de-Andres

**Affiliations:** 1 Internal Medicine Department, Hospital General Universitario Gregorio Marañón, Universidad Complutense de Madrid, Instituto de Investigación Sanitaria Gregorio Marañón (IiSGM), 28007 Madrid, Spain; 2 Department of Public Health and Maternal & Child Health, Faculty of Medicine, Universidad Complutense de Madrid, 28040 Madrid, Spain; 3 Respiratory Care Department, Hospital General Universitario Gregorio Marañón, Universidad Complutense de Madrid, Instituto de Investigación Sanitaria Gregorio Marañón (IiSGM), 28007 Madrid, Spain; 4 Preventive Medicine and Public Health Teaching and Research Unit, Health Sciences Faculty, Universidad Rey Juan Carlos, 28922 Alcorcón, Spain; 5 Department of Nursery and Physiotherapy, Faculty of Medicine and Health Sciences, University of Alcalá, 28801 Alcalá de Henares, Spain

**Keywords:** infective endocarditis, sex, heart valve surgery, comorbidities, in-hospital mortality

## Abstract

(1) Background: A description of the trends and outcomes during hospitalization for infective endocarditis (IE) according to sex. (2) Methods: Using Spanish national hospital discharge data (2016–2020), we built Poisson regression models to compare the age-adjusted time trends for the incidence rate. We used propensity score matching (PSM) to compare the clinical characteristics and the in-hospital mortality (IHM) between men and women hospitalized with IE. (3) Results: We identified 10,459 hospitalizations for IE (33.26% women). The incidence of IE remained stable during this five-year period. The age-adjusted incidence of IE was two-fold higher among men vs. women (IRR = 2.08; 95%CI 2.0–2.17). Before PSM, women with IE were significantly older than men (70.25 vs. 66.24 years; *p* < 0.001) and had lower comorbidity according to the Charlson comorbidity index (mean 1.38 vs. 1.43; *p* = 0.019). After PSM, the IHM among women admitted for IE remained >3 points higher than that among men (19.52% vs. 15.98%; *p* < 0.001). (4) Conclusions: The incidence of IE was two-fold higher among men than among women. IHM was significantly higher among women after accounting for the potential confounders.

## 1. Introduction

Infective endocarditis (IE) has classically been associated with a grim prognosis, with an in-hospital mortality (IHM) ranging from 11% to 20% [1,2]. A deeper understanding of the factors that contribute to worsening the outcomes could inform clinical decisions to improve the management of the patients admitted to the hospital for IE.

Some authors have claimed that sex plays a role in the outcome of patients admitted for IE [3]. Beyond the distinct biological factors possibly underlying sex-related disparities in the host response to the infection, gender could influence patients’ and doctors’ behaviors and thus modify the clinical course of the disease [4]. For instance, lower rates of heart valve replacement surgery among women have been reported during hospitalization for IE [5].

Older research from our country found that the female sex is associated with IHM in IE [6]. However, this research work mainly focused on microbiological isolations and differences among treating hospitals and did not specifically address the effect exerted by sex on mortality. Contrarily, other studies support a trend of a lower IHM among women [7]. Moreover, different researchers have published nonsignificant differences in IHM between both sexes, like the paper by Sevilla T et al. [8]. However, in this study, the IHM was 28% among men vs. 35% among women (*p* value = 0.1), conveying the idea that a lack of statistical power due to small study populations may add confusion. Residual confounding is an important issue concerning randomized clinical trials. Propensity score matching (PSM) might help reduce the impact of unaccounted factors in observational studies [9]. Recent research from our country using PSM has revealed higher mortality among women admitted for IE [10]. However, this work was not fully representative of national data because the registry used for this investigation is integrated by multidisciplinary groups from large academic centers that actively included new IE cases and specifically evaluated the role of surgery in people admitted for IE [10].

With this background, in this investigation, we aimed to describe the incidence of hospitalizations for IE among women and men in Spain for the period 2016–2020, assessing sex differences. We also compared the clinical characteristics, use of therapeutic procedures, and in-hospital outcomes according to sex using PSM. 

## 2. Materials and Methods

### 2.1. Study Design, Study Population, and Data Assessment

We performed an observational, sex-stratified cohort study based on data from the Hospital Discharge Records of the Spanish National Health System (RAE-CMBD, *Registro de Actividad de Atención Especializada-Conjunto Mínimo Básico de Datos* [Register of Specialized Care–Basic Minimum Database]) for the period 1 January 2016 to 31 December 2020. The discharge records were coded based on the International Classification of Disease, Tenth Revision (ICD-10). Details on the RAE-CMBD are available online [11].

The study population comprised every person aged ≥18 years hospitalized with an ICD-10 diagnosis code for IE (I33.0; I33.9; I38) in the first or second diagnostic position in their discharge reports. This method to identify IE hospitalizations has been previously used for research purposes in our country [6].

We excluded patients with missing data for age (*n* = 4), sex (*n* = 6), and discharge destination (*n* = 10). If the same patient was admitted with a diagnosis of IE more than once during the 2016–2020 period, we only considered the first episode in this research. 

The main variables were trends in the incidence of IE in men and women, IHM, and length of hospital stay (LOHS). We also analyzed comorbidities and therapeutic procedures in men and women with IE. Comorbidity was measured using the Charlson comorbidity index (CCI) calculated based on ICD-10 codes, as described elsewhere [12,13].

To calculate the incidence rates, we used the population data provided by the Spanish National Statistics Institute for the years 2016–2020, grouped by age and sex [14].

We reported, for each patient, the following diagnoses: prevalent heart valvulopathy, congenital malformation of the heart, prosthetic valve carrier status, drug abuse, COVID-19, atrial fibrillation, ischemic heart disease, periannular complications/atrioventricular block, septic arterial embolism and shock. As for pathogens, we sought bacteremia by *Staphylococcus*, *Streptococcus*, Gram-negative bacilli, and fungi.

We also collected data on procedures like dialysis, heart valve surgery, mechanical ventilation, and pacemaker implantation. The ICD10 codes used for these diagnoses and procedures are shown in Table S1.

Finally, the hospital department where the patients were admitted was analyzed.

### 2.2. Propensity Score Matching

The PSM method consisted of selecting (for each woman) a man with the same or closest propensity score (PS) obtained with multivariable logistic regression, so we could match the structure of the confounding factors for both sexes. We used year of hospitalization, age, and all the comorbidities present on admission as matching conditions to calculate the PS [15].

The matching method chosen was one-to-one using calipers of width equal to 0.2 of the standard deviation of the logit of the PS. Estimating the absolute standardized difference before and after matching allowed us to assess the quality of the PSM process. Populations are considered to be well balanced whenever the absolute standardized differences were <10% after PSM [15].

### 2.3. Statistical Analysis

We estimated the incidence of IE per man and woman hospitalized for each of the five years analyzed. Age-adjusted incidence rate ratios (IRRs) with their 95% confidence intervals (95% CIs) were calculated using Poisson regression models to compare the incidence of IE according to sex. 

We show the mean and standard deviation (SD) or median and interquartile range (IQR) for the continuous variables and frequency and percentage for the categorical variables. We compared the continuous variables using the t test or the Mann–Whitney test, and categorical variables using the chi-square test.

To assess changes over time, we used Poisson regression for the incidence, Cochran-Armitage tests for categorical variables, and the Jonckheere-Terpstra test for the LOHS.

Multivariable trends in the incidence of IE adjusted by age were evaluated with Poisson regression. We provided the annual percentage change (APC) with 95% confidence interval.

The statistical analysis and the PSM were conducted using Stata version 14 (Stata, College Station, TX, USA), and significance was set at *p* < 0.05 (2-sided).

### 2.4. Ethics

The access to the RAE-CMBD is universal under request (to the Spanish Ministry of Health) [16]. Since this is an anonymous registry, it is not deemed necessary to ask for individual written consent from the patients or to apply for an ethics committee approval, following Spanish legislation.

## 3. Results

We identified a total of 10,459 patients aged ≥18 years with an admission diagnosis of IE in Spain during the period 2016–2020. Women represented 33.26% (*n* = 3479) of the study population.

### 3.1. Incidence of Patients Admitted to Hospitals with IE and Hospital Department of Admission According to Sex

The incidence of IE was significantly higher in men than in women for all the years analyzed (*p* < 0.001), with an age-adjusted IRR of 2.08 (95% CI 2.00–2.17) for men vs. women. As can be seen in Table 1, the crude incidence of IE remained stable from 2016 to 2020 among both men and women.

We could see no significant changes in the incidence of IE over time for women (APC −0.07%; 95% CI, −0.17% to 0.08%; *p*  = 0.458) or men with IE (APC, 0.03%; 95% CI, −0.09% to 0.03%; *p*  =  0.689) in the multivariable regression model.

Over time, the mean age increased only in men (65.53 ±17.31 years in 2016 vs. 67.45 ± 15.24 in 2020; *p* < 0.001). The presence of previous mitral, aortic, and tricuspid valve disease and the mean CCI increased significantly among both sexes. Congenital malformation of the heart remained constant over the study period for both sexes, with figures ranging from 2% to 4%.

LOHS was around 18 days in women and 19 days in men. We found no significant differences in crude IHM among women (19.35% in 2016 vs. 21.81% in 2020; *p* = 0.441) or men (14.37% in 2016 vs. 15.53%; *p* = 0.275) over time (Table 1).

The distributions by hospital departments where patients with IE were admitted according to sex are shown in Table S2. For both sexes, the most common admission department was Internal Medicine, with a significantly higher proportion of women than men (43.32% vs. 38.94%; *p* < 0.001). However, Cardiology (17.98% vs. 16.21%; =0.025), Cardiovascular Surgery (14.15% vs. 9.89%; *p* < 0.01), and Infectious Diseases (10.33% vs. 7.85%; *p* < 0.001) were more frequent among men. No significant differences were found for the Intensive Care Unit (7.27% for women and 7.21% for men).

### 3.2. Clinical Characteristics and Hospital Outcomes for Women and Men Admitted to the Hospital for IE

Before PSM, when all patients hospitalized from 2016 to 2020 were grouped, women with IE were significantly older than men (70.25 vs. 66.24; *p* < 0.001) but had fewer comorbidities according to the CCI (1.38 vs. 1.43; *p* = 0.019) (Table 2). Men suffered from most of the comorbid conditions analyzed more frequently than women. Nonetheless, dementia and atrial fibrillation were more prevalent among women. After PSM, the differences seen between men and women before PSM became nonsignificant.

**Figure 1:**
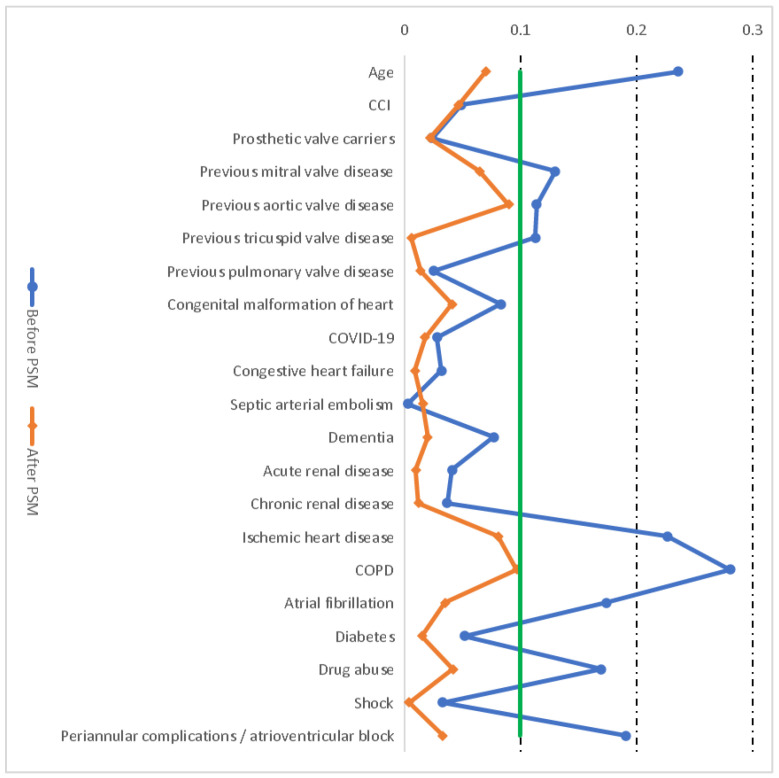
Love plot showing the comparison of covariate values for men and women: absolute standardized differences before and after propensity score matching (PSM). Green line shows the absolute standardized differences of 10%. Dotted lines show 20% and 30% standardized differences.

Shown in Table 2 and Figure 1 are the absolute standardized differences before and after PSM. As can be seen in Figure 1, a significant imbalance could be ruled out since all the absolute standardized differences after PSM were below 10% [15].

In Table 3, we show the distribution of the isolated pathogens, therapeutic procedures, and hospital outcomes among women and men with IE, both before and after PSM. Streptococcus bacteremia was more incident in men, whereas Gram-negative bacilli were more incident in women, even after PSM. Women underwent heart valve surgery and pacemaker implantation less often than men, even after PSM (16.3% and 4.02% vs. 18.74% and 5.29%; *p* = 0.007 and *p* = 0.012, respectively). However, mechanical ventilation was more often coded among women than among men (10.32% vs. 8.88%; *p* = 0.042). IHM among women admitted for IE remained over 3% higher than among men (19.52% vs. 15.98%; *p* < 0.001).

### 3.3. Variables Associated with IHM for Women and Men Admitted to the Hospital with a Diagnosis of IE

We show IHM among women and men with IE before and after PSM according to the prespecified variables in Table 4. Older ages were associated with increased IHM among both sexes. Even after PSM, IHM among women was higher than among men for several conditions, such as previous mitral disease (*p* < 0.001), septic arterial embolism (*p* = 0.032), acute renal disease (*p* = 0.005), atrial fibrillation (*p* = 0.036), diabetes (*p* = 0.004), Gram-positive cocci bacteremia, and heart valve surgery (*p* = 0.01).

## 4. Discussion

Here, we found that the incidence of IE among men doubled the incidence among women. Other studies have also reported higher incidence rates among men vs. women [5,17]. Two recent meta-analyses from one research group, which included European and North American studies, respectively, confirmed the preponderance of male sex among patients admitted for IE [18,19]. The reason for this consistent finding is not clear; it could perhaps be due to recognizable sex-specific predisposing conditions or, eventually, more frequent episodes of low-grade bacteremia among men [20]. It has been proposed that hormonal factors could diminish the incidence of IE among women by protecting them from endothelial damage [21].

In our population, the incidence of IE remained stable over time for both sexes. In this regard, we found conflicting results in the literature [17,19,22,23,24,25,26]. A systematic review by Talha et al. evaluated the population-based incidence of IE in Europe. The pooled regression estimate was a 4.1 ± 1.2% for yearly increments in IE incidence, which translated into a compound increase of 106% over 18 years (2000–2018) [18]. More years of follow-up will be needed to confirm the stabilization of the IE incidence in our country.

The increase from 2016 to 2020 in the prevalence of comorbidities among men and women with IE could partially obey population aging, as previously described in Spain and other countries [6,17,23,24,25]. Besides this increment in the comorbidity, the IHM did not show a significant increase over the study period, and this suggests that the management and pharmacological treatment of IE patients in Spain may be improving [6].

We detected that women underwent heart valve surgery and pacemaker implantation less often than men, whereas they received invasive lung ventilation more often than men, even after PSM. These facts are relevant since pacemaker implantation was associated with lower IHM among both sexes. We cannot dismiss the possibility of reverse causation in this association, as better clinical conditions may have prompted the implantation of the devices in patients prone to better outcomes. When surgery is indicated, failure to perform the operation was associated with the worst prognosis in one study [27,28]. Nevertheless, to make things more complex, other studies had reported worse outcomes for women when they were operated on because of IE [29].

Even after PSM, Streptococcus bacteremia was more incident among men, whereas Gram-negative bacilli bacteremia was more incident among women, in accordance with previous reports [8]. We might hypothesize that men have worse oral hygiene habits than women [30] and, consequently, a higher incidence of Streptococcus viridans bacteremias. Gram-negative bacilli bacteremias might derive from urinary tract infections, which are more common among women. Streptococcus bacteremia was associated with a lower IHM. This is coincidental from the previously published literature, especially when compared with Staphylococcus IE [6,27,31]. A higher incidence on native valves, a better profile of antimicrobial susceptibility, a lower capacity of valve destruction and abscess formation, and less common peripheral embolization could explain this better outcome for Streptococcus IE. 

The odds of dying during hospitalization for IE were higher among women after PSM, which means that this finding is apparently not explained by the remaining variables analyzed. Conflicting results have been reported by previous work that studied the effect of sex on survival after the diagnosis of IE [3,7,10,32]. Varela-Barca et al. [10] communicated a 41% higher IHM among women with IE as compared with men in our country (OR, 1.41; 95% CI 1.21–1.65). Whereas a poorer overall baseline condition among women has been proposed to be responsible for this finding, it has been speculated that women develop heart disease later in life after the hormonal protective effects exerted by estrogens vanish [10,32].

We might theorize about a distinct biological basis or differences in the clinical profile to explain the worse outcomes seen among women, but we are more concerned about a possible gender bias in the clinical management of the condition beyond the measured factors. Physicians’ perception of frailty may differ for female vs. male patients, and this perception might lead to the adoption of comfort measures earlier for female than for male patients, hence driving an unfair limitation on the therapeutic efforts among women [33,34,35]. In fact, in Spain, the higher mortality among women has been linked to different criteria to proceed with heart valve surgery depending on patients’ gender [36]. Furthermore, IE could also be considered more often as a differential diagnosis in men than in women, thus allowing the diagnosis at an earlier stage, which would improve the prognosis. Future investigations should clarify these hypotheses.

In our population, the prevalence of congenital malformation of the heart was low compared to other recent investigations (2–4%) [37]. Van Melle et al. showed that 11% of the cases of IE in their cohort had a congenital malformation of the heart. We might argue that the data used in their registry probably overestimate the true prevalence of congenital malformation of the heart in people with IE since there is probably a selection bias (that registry offered the patients the possibility of being included in the registry after the diagnosis of IE, and perhaps those people aware of their chronic heart condition showed a higher predisposition to be included in the registry) [37]. However, the outcomes reported by Van Melle were better in this subpopulation for congenital malformation of the heart, with results that are in line with our findings [37].

A remarkable result of our investigation is the lower LOHS compared to other studies [6,10,26]. The reported LOHS for IE ranges between 7 and 43 days, with a substantial variation between studies from different countries, depending on the characteristics of the populations analyzed, data sources, and methods used [6,10,26,38,39]. A recent manuscript from Finland reports a median LOHS of 20.0 days in men and 18.0 days in women, which is quite similar to our results [38]. In the US, using data from the Nationwide Readmission Database for those patients who survived hospitalization for IE, the median length of stay was 10 (IQR, 6–17) days: much shorter than our results (18 days) [39]. Our data are from very recent years, and these figures probably reflect earlier diagnoses, more aggressive clinical management of the patients, and better results from surgery. However, future studies are needed to explain the differences in the LOHS reported.

The large sample size of this study, which includes data from 10,459 recent episodes of IE and the widespread coverage of the Spanish population by the RAE-CMBD (>95% of all hospital admissions), gives robustness to our results. However, some limitations should be pointed out. First, our data source was the RAE-CMBD, an administrative database that depends on the information that physicians include in the discharge report and on manual coding on behalf of administrative staff. Second, to our knowledge, the ICD-10 codes for IE in the RAE-CMBD have not been validated so far. However, the results from previous studies conducted in other countries using the International Classification of Disease, Ninth Revision, (ICD-9 and ICD-10) codes in hospital discharge databases suggested good accuracy for the detection of IE cases [24,25,40,41,42,43]. Third, it is unlikely that PSM could fully eliminate residual confounding. Fourth, we excluded 20 patients from the sample (<0.2%) due to missing data, though we believe that a selection bias that could impact the results is improbable. Fifth, the RAE-CMBD only collects information on the diagnosis and procedures for each patient during the hospitalization, but not the dates for each of these diagnoses nor the duration of the symptoms before hospitalization; therefore, it is not possible to calculate the time from the beginning of the symptoms to the diagnosis of IE. Sixth, it is common practice to admit every single case of IE to the hospital when it is the suspected diagnosis at admission. However, most of the cases were probably not suspected at admission but were diagnosed during the hospitalization period. For the latter category, fever, new-onset heart failure, or a general deterioration in the clinical status may have indicated the hospital admission. We cannot rule out some heterogeneity in the clinical presentation of the disease according to sex, but unfortunately, the initial reason for hospital admission was not collected in the database used. Furthermore, in our opinion, the sex differences found in the hospital department where patients were admitted may be justified by the differences in the symptoms of IE when they were admitted to the hospital, the comorbid conditions, and by the higher age of the women. The RAE-CMBD database is also limited by the lack of data on microbiological resistance patterns and the lack of information for identifying the foci of the pathogens isolated or if the IE is device-related. Future studies with more detailed clinical data should include these variables to assess sex differences in IE. Seventh, even if five years may be a short period of time to show a well-defined trend, we used data from 2016 onward because during that year, the RAE-CMBD moved from the ICD 9 to the ICD 10, and the effect of this change in the coding method could affect our results. Finally, the results of this study do not necessarily reflect the actual data from other countries.

## 5. Conclusions

Hospital admission for IE in adults in Spanish hospitals during the period 2016–2020 was more frequent among men than among women. The in-hospital mortality among those women admitted for IE was significantly higher than that among men. We observed a lower rate of invasive cardiac procedures among women admitted for IE. These and other factors should be better characterized to minimize the differences in mortality between the sexes for people admitted for IE.

## Figures and Tables

**Table 1:** Incidence, clinical characteristics, and in-hospital outcomes of patients hospitalized with infective endocarditis in Spain from 2016 to 2020 according to sex.

		2016	2017	2018	2019	2020	*p*-Value *
N, (incidence per 100,000 people per year)	Both sexes	1975 (4.25)	2090 (4.49)	2242 (4.8)	2222 (4.72)	1930 (4.08)	0.656
N, (incidence per 100,000 people per year)	Women	646 (2.73)	704 (2.97)	772 (3.24)	715 (2.98)	642 (2.66)	0.672
	Men	1329 (5.83)	1386 (6.07)	1470 (6.42)	1507 (6.53)	1288 (5.55)	0.826
Age, mean (SD)	Women	70.21 (18.21)	69.00 (19.34)	69.88 (18.27)	70.53 (16.74)	71.78 (14.77)	0.064
	Men	65.53 (17.31)	64.62 (17.18)	67.23 (15.82)	66.36 (15.90)	67.45 (15.24)	<0.001
CCI index, mean (SD)	Women	1.27 (1.13)	1.35 (1.12)	1.30 (1.11)	1.46 (1.18)	1.50 (1.17)	<0.001
	Men	1.30 (1.19)	1.43 (1.21)	1.49 (1.25)	1.43 (1.25)	1.53 (1.27)	<0.001
Prosthetic valve carriers, n (%)	Women	69 (10.68)	68 (9.66)	65 (8.42)	55 (7.69)	65 (10.12)	0.287
	Men	121 (9.10)	117 (8.44)	136 (9.25)	121 (8.03)	103 (8.00)	0.647
Previous mitral valve disease, n (%)	Women	195 (30.19)	189 (26.85)	235 (30.44)	244 (34.13)	217 (33.80)	0.021
	Men	311 (23.40)	349 (25.18)	370 (25.17)	364 (24.15)	367 (28.49)	0.032
Previous aortic valve disease, n (%)	Women	128 (19.81)	163 (23.15)	174 (22.54)	206 (28.81)	177 (27.57)	<0.001
	Men	357 (26.86)	410 (29.58)	420 (28.57)	444 (29.46)	422 (32.76)	0.020
Previous tricuspid valve disease, n (%)	Women	43 (6.66)	54 (7.67)	90 (11.66)	82 (11.47)	74 (11.53)	0.001
	Men	66 (4.97)	80 (5.77)	101 (6.87)	111 (7.37)	112 (8.70)	0.002
Previous pulmonary valve disease, n (%)	Women	3 (0.46)	7 (0.99)	2 (0.26)	2 (0.28)	3 (0.47)	0.268
	Men	3 (0.23)	7 (0.51)	4 (0.27)	2 (0.13)	7 (0.54)	0.244
Congenital malformation of heart, n (%)	Women	13 (2.01)	25 (3.55)	28 (3.63)	20 (2.8)	19 (2.96)	0.403
	Men	44 (3.31)	51 (3.68)	57 (3.88)	55 (3.65)	40 (3.11)	0.820
Drug abuse, n (%)	Women	10 (1.55)	13 (1.85)	8 (1.04)	5 (0.70)	5 (0.78)	0.208
	Men	44 (3.31)	56 (4.04)	68 (4.63)	58 (3.85)	41 (3.18)	0.274
LOHS, median (IQR)	Women	16.5 (27)	17 (28)	18 (25)	19 (24)	18 (24)	0.681
	Men	20 (25)	19 (26)	19 (26)	19 (25)	19 (23)	0.897
IHM, n (%)	Women	125 (19.35)	128 (18.18)	142 (18.39)	144 (20.14)	140 (21.81)	0.441
	Men	191 (14.37)	183 (13.20)	232 (15.78)	233 (15.46)	200 (15.53)	0.275

* *p* value for time trend. SD: standard deviation; CCI: Charlson comorbidity index; LOHS: length of hospital stay; IQR: interquartile range; IHM: in-hospital mortality.

**Table 2:** Distribution of clinical characteristics of women and men with infective endocarditis in Spain (2016–20), before and after propensity score matching (PSM).

	BEFORE PSM				AFTER PSM			
	Women	Men	ASD	*p*-Value	Women	Men	ASD	*p*-Value
N	3479	6980	NA	NA	3479	3479	NA	NA
Age, mean (SD)	70.25 (17.59)	66.24 (16.33)	0.236	<0.001	70.25 (17.59)	70.11 (11.87)	0.07	0.697
CCI index, mean (SD)	1.38 (1.15)	1.43 (1.24)	0.049	0.019	1.38 (1.15)	1.42 (1.11)	0.047	0.140
Prosthetic valve carriers, n (%)	322 (9.26)	598 (8.57)	0.024	0.242	322 (9.26)	344 (9.89)	0.022	0.370
Previous mitral valve disease, n (%)	1080 (31.04)	1761 (25.23)	0.13	<0.001	1080 (31.04)	1069 (30.73)	0.065	0.781
Previous aortic valve disease, n (%)	848 (24.37)	2053 (29.41)	0.114	<0.001	848 (24.37)	789 (22.69)	0.09	0.097
Previous tricuspid valve disease, n (%)	343 (9.86)	470 (6.73)	0.113	<0.001	343 (9.86)	337 (9.69)	0.006	0.809
Previous pulmonary valve disease, n (%)	17 (0.49)	23 (0.33)	0.025	0.214	17 (0.49)	20 (0.57)	0.014	0.621
Congenital malformation of heart, n (%)	105 (3.02)	247 (3.54)	0.083	0.164	105 (3.02)	87 (2.52)	0.041	0.705
COVID-19, n (%)	20 (0.57)	31 (0.44)	0.028	0.366	20 (0.57)	27 (0.78)	0.018	0.306
Congestive heart failure, n (%)	392 (11.27)	717 (10.27)	0.032	0.119	392 (11.27)	382 (10.98)	0.009	0.703
Septic arterial embolism, n (%)	150 (4.31)	305 (4.37)	0.003	0.891	150 (4.31)	161 (4.63)	0.016	0.523
Dementia, n (%)	92 (2.64)	108 (1.55)	0.077	<0.001	92 (2.64)	82 (2.36)	0.02	0.443
Acute renal disease, n (%)	690 (19.83)	1500 (21.49)	0.041	0.050	690 (19.83)	676 (19.43)	0.01	0.673
Chronic renal disease, n (%)	640 (18.4)	1186 (16.99)	0.037	0.075	640 (18.40)	656 (18.86)	0.012	0.622
Ischemic heart disease, n (%)	343 (9.86)	1229 (17.61)	0.227	<0.001	343 (9.86)	379 (10.90)	0.081	0.156
COPD, n (%)	104 (2.99)	683 (9.79)	0.281	<0.001	104 (2.99)	131 (3.77)	0.097	0.072
Atrial fibrillation, n (%)	1167 (33.54)	1789 (25.63)	0.174	<0.001	1167 (33.54)	1223 (35.15)	0.035	0.157
Diabetes, n (%)	816 (23.46)	1792 (25.67)	0.052	0.013	816 (23.46)	793 (22.79)	0.015	0.513
Drug abuse, n (%)	41 (1.18)	267 (3.83)	0.17	<0.001	41 (1.18)	60 (1.73)	0.042	0.057
Shock, n (%)	65 (1.87)	163 (2.34)	0.033	0.123	65 (1.87)	63 (1.81)	0.004	0.858
Periannular complications/atrioventricular block, n (%)	142 (4.08)	425 (6.09)	0.191	<0.001	142 (4.08)	122 (3.51)	0.033	0.209

NA: not applicable; SD: standard deviation; CCI: Charlson comorbidity index; COPD: chronic obstructive pulmonary disease. ASD: absolute standardized differences.

**Table 3:** Distribution of isolated pathogens, therapeutic procedures, and hospital outcomes among women and men with infective endocarditis in Spain (2016–2020), before and after propensity score matching (PSM).

	BEFORE PSM			AFTER PSM		
	Women	Men	*p*-Value	Women	Men	*p*-Value
Staphylococcus bacteremia, n (%)	992 (28.51)	2035 (29.15)	0.496	992 (28.51)	978 (28.11)	0.709
Streptococcus bacteremia, n (%)	705 (20.26)	1715 (24.57)	<0.001	705 (20.26)	848 (24.37)	<0.001
Gram-negative bacilli bacteremia, n (%)	353 (10.15)	459 (6.58)	<0.001	353 (10.15)	240 (6.90)	<0.001
Fungemia, n (%)	15 (0.43)	34 (0.49)	0.693	15 (0.43)	14 (0.40)	0.852
Heart valve surgery n (%)	567 (16.30)	1560 (22.35)	<0.001	567 (16.30)	652 (18.74)	0.007
Dialysis, n (%)	172 (4.94)	350 (5.01)	0.876	172 (4.94)	141 (4.05)	0.073
Pacemaker implantation, n (%)	140 (4.02)	385 (5.52)	0.001	140 (4.02)	184 (5.29)	0.012
Mechanical ventilation, n (%)	359 (10.32)	788 (11.29)	0.135	359 (10.32)	309 (8.88)	0.042
LOHS, median (IQR)	18 (25)	19 (25)	0.085	18 (25)	19 (25)	0.271
IHM, n (%)	679 (19.52)	1039 (14.89)	<0.001	679 (19.52)	556 (15.98)	<0.001

LOHS: length of hospital stay; IQR: interquartile range; IHM: in-hospital mortality. Heart valve surgery included aortic, mitral, tricuspid, and pulmonary.

**Table 4:** In hospital mortality according to study variables of women and men with infective endocarditis in Spain (2016–2020), before and after propensity score matching (PSM).

	BEFORE PSM			AFTER PSM		
	Women	Men	*p*-Value	Women	Men	*p*-Value
N	679	1039	NA	679	556	NA
Age, mean (SD)	75.94 (11.72)	72.88 (12.03)	<0.001	75.94 (11.72)	75.97 (10.12)	0.966
<40 years old, n (%)	4 (1.71)	10 (2.28)	0.621	4 (1.71)	0 (0)	NA
40–66 years old, n (%)	121 (15.37)	273 (10.62)	<0.001	121 (15.37)	101 (10.58)	0.003
67–75 years old, n (%)	151 (18.48)	262 (15.35)	0.047	151 (18.48)	139 (14.32)	0.018
≥76 years old, n (%)	403 (24.56)	494 (21.82)	0.045	403 (24.56)	316 (20.80)	0.012
CCI index, mean (SD)	1.81 (1.16)	2.01 (1.25)	0.001	1.81 (1.16)	1.72 (1.15)	0.163
Prosthetic valve carriers, n (%)	61 (18.94)	79 (13.21)	0.022	61 (18.94)	48 (13.95)	0.083
Previous mitral valve disease, n (%)	227 (21.02)	260 (14.76)	<0.001	227 (21.02)	173 (14.65)	<0.001
Previous aortic valve disease, n (%)	175 (20.64)	342 (16.66)	0.011	175 (20.64)	142 (20.97)	0.872
Previous tricuspid valve disease, n (%)	73 (21.28)	67 (14.26)	0.009	73 (21.28)	52 (15.43)	0.050
Previous pulmonic valve disease, n (%)	2 (11.76)	1 (4.35)	0.397	2 (11.76)	1 (5.00)	0.465
Congenital malformation of heart, n (%)	7 (6.67)	4 (5.67)	0.718	7 (6.67)	4 (7.55)	0.832
COVID-19, n (%)	4 (20.00)	7 (22.58)	0.827	4 (20.00)	7 (25.93)	0.636
Congestive heart failure, n (%)	103 (26.28)	168 (23.43)	0.292	103 (26.28)	100 (26.18)	0.975
Septic arterial embolism, n (%)	52 (34.67)	58 (19.02)	<0.001	52 (34.67)	38 (23.60)	0.032
Dementia, n (%)	24 (26.09)	28 (25.93)	0.979	24 (26.09)	19 (23.17)	0.656
Acute renal disease, n (%)	256 (37.10)	442 (29.47)	<0.001	256 (37.10)	202 (29.88)	0.005
Chronic renal disease, n (%)	172 (26.88)	275 (23.19)	0.081	172 (26.88)	158 (24.09)	0.249
Ischemic heart disease, n (%)	89 (25.95)	230 (18.71)	0.003	89 (25.95)	25 (17.36)	0.042
COPD, n (%)	30 (28.85)	141 (20.64)	0.060	30 (28.85)	1 (20.00)	0.671
Atrial fibrillation, n (%)	269 (23.05)	368 (20.57)	0.109	269 (23.05)	239 (19.54)	0.036
Diabetes, n (%)	183 (22.43)	296 (16.52)	<0.001	183 (22.43)	133 (16.77)	0.004
Drug Abuse, n (%)	5 (12.20)	16 (5.99)	0.151	5 (12.20)	6 (9.38)	0.646
Shock, n (%)	45 (69.23)	89 (54.60)	0.044	45 (69.23)	33 (52.38)	0.052
Periannular complications/atrioventricular block, n (%)	30 (21.13)	83 (19.53)	0.680	30 (21.13)	39 (18.40)	0.525
Staphylococcus bacteremia, n (%)	274 (27.62)	420 (20.64)	<0.001	274 (27.62)	225 (23.01)	0.019
Streptococcus bacteremia, n (%)	75 (10.64)	118 (6.88)	0.002	75 (10.64)	65 (7.67)	0.042
Gram-negative bacteremia, n (%)	72 (20.40)	102 (22.22)	0.530	72 (20.40)	52 (21.67)	0.709
Fungemia, n (%)	6 (40.00)	15 (44.12)	0.788	6 (40.00)	9 (64.29)	0.196
Heart valve surgery, n (%)	130 (22.93)	253 (16.22)	<0.001	130 (22.93)	111 (17.02)	0.010
Dialysis, n (%)	81 (47.09)	131 (37.43)	0.035	81 (47.09)	54 (38.30)	0.119
Pacemaker implantation, n (%)	17 (12.14)	37 (9.61)	0.399	17 (12.14)	17 (9.24)	0.400
Mechanical ventilation, n (%)	152 (42.34)	278 (35.28)	0.022	152 (42.34)	111 (35.92)	0.091
LOHS, Median (IQR)	14 (21)	15 (21)	0.855	14 (21)	16 (21)	0.529

NA: not applicable; SD: standard deviation; CCI: Charlson comorbidity index; COPD: chronic obstructive pulmonary disease LOHS: length of hospital stay; IHM: in-hospital mortality. Heart valve surgery included aortic, mitral, tricuspid, and pulmonary.

## Data Availability

According to the contract signed with the Spanish Ministry of Health and Social Services, which provided access to the databases from the Spanish National Hospital Database (RAE-CMBD, *Registro de Actividad de Atención Especializada. Conjunto Mínimo Básico de Datos*, Registry of Specialized Health Care Activities. Minimum Basic Data Set), we cannot share the databases with any other investigator, and we have to destroy the databases once the investigation has concluded. Consequently, we cannot upload the databases to any public repository. However, any investigator can apply for access to the databases by filling out the questionnaire available at http://www.msssi.gob.es/estadEstudios/estadisticas/estadisticas/estMinisterio/SolicitudCMBDdocs/Formulario_Peticion_Datos_CMBD.pdf. All other relevant data are included in the paper. (accessed on 21 September 2022).

## Supplementary Materials

The following supporting information can be downloaded at: https://www.mdpi.com/article/10.3390/jcm11226847/s1, Table S1: Diagnosis, procedures, and pathogens analyzed with their corresponding ICD10 codes. Table S2: Hospital departments where patients with infective endocarditis were admitted according to sex. Hospital Discharge Records of the Spanish National Health System (RAE-CMBD) from 2016 to 20.
