# Supplementary material for: Differences in Sex and the Incidence and In-Hospital Mortality among People Admitted for Infective Endocarditis in Spain, 2016–2020
Source: J Clin Med. 2022 Nov 20;11(22):6847. doi: 10.3390/jcm11226847 (PMC9698698; doi:10.3390/jcm11226847)
Supplement: Supplementary file 1 [file jcm-11-06847-s001.zip › jcm-1980476-supplementary.pdf]

Table S1. Diagnosis, procedures and pathogens analyzed with their corresponding ICD10 codes.

Diagnosis/Procedures/Pathogens	ICD-10 codes
Previous mitral valve disease	I050; I051; I052; I058; I059; I080; I081; I083; I340; I341; I342; I348; I349
Previous aortic valve disease	I060; I061; I062; I068; I069; I080; I350; I351; I352; I358; I359; I082; I083
Previous tricuspid valve disease	I070; I071; I072; I078; I079; I081; I082; I083; I360; I361; I362; I368; I369
Previous pulmonary valve disease	I370; I371; I372; I378; I379
Congenital malformation of heart	Q20.x; Q21.x; Q22.x; Q23.x; Q24.x
Drug abuse	F1110; F1111; F11120; F11121; F11122; F11129; F1114; F11150; F11151; F11159; F11181; F11182; F11188; F1119; F1120; F1121; F11220; F11221; F11222; F11229; F1123; F1124; F11250; F11251; F11259; F11281; F11282; F11288; F1129; F1210; F1211; F12120; F12121; F12122; F12129; F12150; F12151; F12159; F12180; F12188; F1219; F1220; F1221; F12220; F12221; F12222; F12229; F1223; F12250; F12251; F12259; F12280; F12288; F1229; F1310; F1311; F13120; F13121; F13129; F1314; F13150; F13151; F13159; F13180; F13181; F13182; F13188; F1319; F1320; F1321; F13220; F13221; F13229; F13230; F13231; F13232; F13239; F1324; F13250; F13251; F13259; F1326; F1327; F13280; F13281; F13282; F13288; F1329; F1410; F1411; F14120; F14121
COVID-19	B34.2; B97.29; U07.1
Atrial fibrillation	I48.0; I48.1; I48.2; I48.91
Ischemic heart disease	I20-I25
Periannular complications/atrioventricular block	I51.1; I51.2; I44.2; I44.1
Septic arterial embolism	I76
Shock	R57.0
Prosthetic valve carriers	Z95.2
Dialysis	5A1D xxx
Heart valve surgery (aortic, mitral, tricuspid, pulmonary)	024F07J; 024F08J; 024F0JJ; 024F0KJ; 02BF0ZX; 02BF0ZZ; 02CF0ZZ; 02NF0ZZ; 02QF0ZJ; 02QF0ZZ; 02RF07Z; 02RF08Z; 02RF0JZ; 02RF0KZ; 02UF07J; 02UF07Z; 02UF08J; 02UF08Z; 02UF0JJ; 02UF0JZ; 02UF0KJ; 02UF0KZ; 02WF07Z; 02WF08Z; 02WF0JZ; 02WF0KZ; X2RF032<br>024G072; 024G082; 024G0J2; 024G0K2; 02BG0ZX; 02BG0ZZ; 02CG0ZZ; 02NG0ZZ; 02QG0ZE; 02QG0ZZ; 02RG07Z; 02RG08Z; 02RG0JZ; 02RG0KZ; 02UG07E; 02UG07Z; 02UG08E; 02UG08Z; 02UG0JE; 02UG0JZ; 02UG0KE; 02UG0KZ; 02VG0ZZ; 02WG07Z; 02WG08Z; 02WG0JZ; 02WG0KZ<br>024J072; 024J082; 024J0J2; 024J0K2; 02BJ0ZX; 02BJ0ZZ; 02CJ0ZZ; 02NJ0ZZ; 02QJ0ZG; 02QJ0ZZ; 02RJ07Z; 02RJ08Z; 02RJ0JZ; 02RJ0KZ; 02UJ07G; 02UJ07Z; 02UJ08G; 02UJ08Z; 02UJ0JG; 02UJ0JZ; 02UJ0KG; 02UJ0KZ; 02WJ07Z; 02WJ08Z; 02WJ0JZ; 02WJ0KZ<br>02BH0ZX; 02BH0ZZ; 02CH0ZZ; 02NH0ZZ; 02QH0ZZ; 02RH07Z; 02RH08Z; 02RH0JZ; 02RH0KZ; 02TH0ZZ; 02UH07Z; 02UH08Z; 02UH0JZ; 02UH0KZ; 02WH07Z; 02WH08Z; 02WH0JZ; 02WH0KZ
Mechanical ventilation	5A09357; 5A09457; 5A09557; 5A1945Z; 5A1955Z; 5A1935Z
Pacemaker implantation	02HKxxx; 02HLxxx; 02H4xxx; 02H6xxx; 02H7xxx; 02HNxxx; 0JH6xxx; 0JH8xxx
Staphylococcus bacteremia	A4101; A4102; A411; A412; A4901; A4902; B9561; B9562; B957; B958
Streptococcus bacteremia	A400; A401; A403; A408; A409; A491; B950; B951; B953; B954; B955
Gram-negative bacilli bacteremia	A413; A4150; A4151; A4152; A4153; A4159; B961; B9620; B9621; B9622; B9623; B9629; B963; B964; B965
Fungemia	B376; B377; B409; B393; B394; B395; B399; B449

The ICD 10 codes for conditions included in the Charlson Comorbidity Index can be found in references: Sundararajan V, Henderson T, Perry C, Muggivan A, Quan H, Ghali WA. New ICD-10 version of the Charlson comorbidity index predicted in-hospital mortality. J Clin Epidemiol. 2004; 57:1288–94. doi: 10.1016/j.jclinepi.2004.03.012.Quan H, Sundararajan V, Halfon P, Fong

A, Burnand B, Luthi JC, et al. Coding algorithms for defining comorbidities in ICD-9-CM and ICD-10 administrative data. Med Care. 2005; 43:1130-9. doi: 10.1097/01.mlr.0000182534.19832.83.

Table S2. Hospital departments where patients with infective endocarditis were admitted according to sex. Hospital Discharge Records of the Spanish National Health System (RAE-CMBD) from 2016 to 20.

**Hospital department**	**Women**		**Men**		**p-value**
	N	%	N	%	
Internal Medicine	1507	43.32	2718	38.94	<0.001
Cardiology	564	16.21	1255	17.98	0.025
Cardiovascular Surgery	344	9.89	988	14.15	<0.001
Infectious diseases	273	7.85	721	10.33	<0.001
Intensive Care Unit	253	7.27	503	7.21	0.902

P value for the comparison between women and men using Chi square test.
